# Nitrogen single-breath washout test for evaluating exercise tolerance and quality of life in patients with chronic obstructive pulmonary disease

**DOI:** 10.1590/1414-431X20177059

**Published:** 2018-02-26

**Authors:** C.A.L. Deus, P.S. Vigário, F.S. Guimarães, A.J. Lopes

**Affiliations:** 1 Programa de Pós-Graduação em Ciências da Reabilitação, Centro Universitário Augusto Motta, Rio de Janeiro, RJ, Brasil; 2 Programa de Pós-Graduação em Ciências Médicas, Faculdade de Ciências Médicas, Universidade do Estado do Rio de Janeiro, Rio de Janeiro, RJ, Brasil

**Keywords:** Chronic obstructive pulmonary disease, Ventilatory efficiency, Pulmonary function tests, Cardiopulmonary exercise testing, Quality of life

## Abstract

Pulmonary function tests (PFTs) traditionally used in clinical practice do not accurately predict exercise intolerance in patients with chronic obstructive pulmonary disease (COPD). The aim of this study was to assess whether the nitrogen single-breath washout (N_2_SBW) test explains exercise intolerance and poor quality of life in stable COPD patients. This cross-sectional study included 31 patients with COPD subjected to PFTs (including the N_2_SBW test) and a cardiopulmonary exercise test (CPET). Patients were also evaluated using the following questionnaires: the COPD assessment test (CAT), the 36-Item Short Form Health Survey (SF36) and St. George's Respiratory Questionnaire (SGRQ). Peak oxygen uptake (peak VO_2_) was negatively correlated with the phase III slope of the N_2_SBW (SIII_N2_) (r=-0.681, P<0.0001) and positively correlated with forced expiratory volume in one second (FEV_1_; r=0.441, P=0.013). Breathing reserve was negatively correlated with SIII_N2_, closing volume/vital capacity, and residual volume (RV) (r=-0.799, P<0.0001; r=-0.471, P=0.007; r=-0.401, P=0.025, respectively) and positively correlated with FEV_1_, forced vital capacity (FVC) and FEV_1_/FVC (r=0.721; P<0.0001; r=0.592, P=0.0004; r=0.670, P<0.0001, respectively). SIII_N2_ and CAT were independently predictive of VO_2_ and breathing reserve at peak exercise. RV, FVC, and FEV_1_ were independently predictive of the SF36-physical component summary, SF36-mental component summary, and breathing reserve, respectively. The SGRQ did not present any independent variables that could explain the model. In stable COPD patients, inhomogeneity of ventilation explains a large degree of exercise intolerance assessed by CPETs and, to a lesser extent, poor quality of life.

## Introduction

Chronic obstructive pulmonary disease (COPD) is a major public health problem worldwide that affects health-related quality of life (HRQoL) of individuals and causes disability and high morbidity and mortality (1). The HRQoL of patients with COPD is impaired by the symptoms and systemic manifestations of the disease, which worsen with disease chronicity and lead to morbidity (2). Moreover, functional disability strongly affects HRQoL of patients with COPD. However, few studies have evaluated the association among the degree of physical activity limitation, the performance of activities of daily living (ADLs), lung function and performance during exercise (3).

Pulmonary function tests (PFTs) are valuable tools for the diagnosis and assessment of the severity of COPD and have many clinical applications, such as evaluating prognosis and response to therapy (1). Spirometry is the most commonly used functional test in the evaluation and management of these patients, despite the limitation that forced expiratory volume in one second (FEV_1_) is a marker of airflow obstruction in the proximal airways, whereas airflow obstruction in COPD occurs primarily in the distal airways (4,5). In recent decades, there have been significant technological advances in tools used to measure lung function. Despite the technological sophistication of traditional functional assessment methods, new PFTs are gradually being incorporated into clinical practice (6). There has been growing interest in the use of the single-breath washout (N_2_SBW) test because it is a simple, non-invasive tool and can detect heterogeneity in the ventilation distribution and small airway disease in cases in which traditional PFTs indicate normal values (7). Increased values of the phase III slope of N_2_SBW (SIII_N2_) and closing volume/vital capacity (CV/VC) indicate heterogeneity in the ventilation distribution and small airway disease, respectively (7). Two recent studies that used the walking test found that the increase in SIII_N2_ was correlated with worsening dyspnea and reduction of the 6-min walking distance in patients with COPD (8,9).

The cardiopulmonary exercise test (CPET) can detect abnormalities in the functional capacity of the respiratory and cardiovascular systems earlier than other tests (10). In patients with COPD, a reduced peak oxygen uptake (peak VO_2_) and a low breathing reserve have been identified as the main contributors that limit exercise (11–13). Difficulty breathing and exercise intolerance have been associated with excessive ventilatory responses to exercise in these patients, even among those with a relatively well-preserved FEV1 (11,12). In fact, the PFTs traditionally used in clinical practice do not accurately predict exercise intolerance, and CPET has been proposed as the gold standard method to evaluate exercise intolerance in patients with COPD (5,13).

As the outcomes of traditional PFTs do not correlate well with CPET parameters, it is important to investigate other pulmonary function methods that are better associated with exercise intolerance in patients with COPD. Considering that ventilatory limitation aggravated by exercise is a major contributor to the reduced functional capacity in COPD patients, we hypothesized that heterogeneity in the ventilation distribution and small airway disease may contribute to better understand exercise intolerance in these patients (10). Thus, the objective of this study was to assess whether parameters of the N_2_SBW test explain exercise intolerance in stable COPD patients. Secondly, we sought to evaluate whether parameters of the N_2_SBW test explain poor quality of life of these patients.

## Patients and Methods

### Patients

Between April 2015 and January 2017, we conducted a cross-sectional study involving 38 consecutive patients with COPD recruited at the Policlínica Newton Bethlem, Rio de Janeiro, Brazil. The exclusion criteria were age less than 40 years, smoking load of fewer than 10 pack-years, previous pleuropulmonary disease not related to COPD, use of supplemental oxygen therapy, previous thoracic surgery, respiratory exacerbations or infections in the past 4 weeks and any comorbid condition that might reduce exercise capacity. The project was approved by the Research Ethics Committee of the Centro Universitário Augusto Motta under CAAE No. 52885116.6.0000.5235 and complied with the Declaration of Helsinki. All subjects signed a consent form.

### Procedures

#### Modified Medical Research Council (m-MRC) scale

The m-MRC scale is a subjective tool that assesses limitations in ADLs and has been validated for use in Portuguese language (14). The m-MRC is a 5-item scale with the following categories: 0 (experiencing shortness of breath only with strenuous exercise); 1 (experiencing shortness of breath when hurrying on level ground or ascending a gentle slope); 2 (walking slower than other people their age due to shortness of breath or having to stop for breath when walking slowly); 3 (stopping for breath after walking less than 100 m or after a few minutes); and 4 (experiencing so much shortness of breath that prevents leaving the home or experiencing shortness of breath when dressing). The m-MRC scale is easy to use: the patient simply selects the item that corresponds to the degree to which dyspnea limits his or her ADLs (1,14).

#### COPD assessment test (CAT) questionnaire

The CAT is a specific questionnaire used to measure the HRQoL of patients with COPD. This one-dimensional measure contains 8 items related to the impairment of health status. At the end of the test, the scores of all the responses are summed, and the clinical impact of COPD is evaluated according to the stratification of CAT scores. The total score ranges from 0 to 40, and higher scores indicate poorer health status (15).

#### ABCD assessment tool

Based on the m-MRC scale, CAT questionnaire, and exacerbation history (0 or 1 not leading to hospital admission *vs* ≥2 not leading to hospital admission or ≥1 leading to hospital admission), patients were classified as follows: A (m-MRC 0–1, CAT <10, and exacerbation history of 0 or 1 not leading to hospital admission); B (m-MRC ≥2, CAT ≥10, and exacerbation history of 0 or 1 not leading to hospital admission); C (m-MRC 0–1, CAT <10, and ≥2 exacerbation histories not leading to hospital admission or ≥1 exacerbation history leading to hospital admission), and D (m-MRC ≥2, CAT ≥10 and ≥2 exacerbation histories not leading to hospital admission or ≥1 exacerbation history leading to hospital admission) (1).

#### Thirty-six-item short form health survey (SF36)

The SF36 is a generic self-applied questionnaire consisting of 36 questions divided into eight areas, which in turn can be grouped into 2 broad categories: a physical component summary (PCS) and a mental component summary (MCS). The scores range from 0 to 100, with higher scores indicating better HRQoL (16).

#### St. George's respiratory questionnaire (SGRQ)

The SGRQ is a validated, well-known self-administered tool that measures symptoms, ADLs, and HRQoL of patients with chronic respiratory diseases, particularly COPD. It consists of 50 items divided into 3 domains: symptoms (8 items), activity (16 items) and impact (26 items). Each item has a derived weight, and lower values indicate better HRQoL (17).

#### Resting lung function

Spirometry and body plethysmography were conducted using Collins Plus Pulmonary Function Testing Systems (Warren E. Collins, Inc., USA) in the Hospital Universitário Pedro Ernesto of the Universidade do Estado do Rio de Janeiro, Brazil, following the standardization of the American Thoracic Society (18). The reference values used were the Brazilian values, and the results are as percentages of the predicted values (19,20). The severity of airflow limitation was classified based on FEV_1_ values after salbutamol use as follows: GOLD 1 (mild, FEV_1_ ≥80% predicted); GOLD 2 (moderate, 50% ≤ FEV_1_ <80% predicted); GOLD 3 (severe, 30% ≤ FEV_1_ <50% predicted), and GOLD 4 (very severe, FEV_1_ <30% predicted) (1).

In addition, the patients underwent N_2_SBW test with a 3000 HDpft (nSpire Health, Inc., USA) according to the recommendations of the American Thoracic Society/European Respiratory Society (7). Briefly, individuals exhaled until reaching the residual volume (RV) and then inhaled 100% O_2_ until reaching total lung capacity (TLC). Then, patients exhaled slowly at a flow rate of approximately 0.3–0.5 L/s until reaching the RV. The concentration of exhaled N_2_ was recorded by a device located at the airway opening. Two indices derived from this procedure were reported as a percentage of the predicted values: SIII_N2_, which is the change in N_2_ concentration between 25 and 75% of the expiratory volume, and the CV/VC ratio, which is the percentage of the VC that is exhaled after the beginning of airway closure (21).

#### Cardiopulmonary exercise test (CPET)

The patients performed the CPET on a treadmill (Inbramed, ATL, Brazil) coupled with a metabolic analyzer (MedGraphics VO2000, Medical Graphics, Inc., USA) in the Laboratório de Movimento Humano at the Centro Universitário Augusto Motta according to previous recommendations (22). We used the ramp protocol, in which the slope and load were individualized by adapting to the capacity of each individual using an optimal period of exercise intensity estimated between 8 and 12 min. Oxygen saturation, heart reserve (HR), 12-lead electrocardiogram tracings, and blood pressure were monitored continuously and measured every 2 min throughout the exercise period. Oxygen uptake (VO_2_), carbon dioxide output (VCO_2_), minute ventilation (VE) and related variables were calculated breath-by-breath (10). Breathing reserve was calculated as the difference between the maximum voluntary ventilation (MVV) at rest and the peak minute ventilation (VE) and was reported as a percentage of MVV [1 – (VE/MVV) x 100] (23). Heart rate reserve (HRR) was calculated as the difference between the heart rates obtained at rest and during peak exercise [(220 – age) – peak HR] (10). The equations for the predicted Brazilian values were used to interpret the results (24).

### Statistical analysis

The Shapiro-Wilk test was used to verify the normality of variables. Results are reported as means±SD or frequencies (percentages). The associations between the variables peak VO_2_, breathing reserve, CAT, SF36 PCS, SF 36 MCS, and SGRQ with demographic, clinical and resting lung function variables were analyzed using Pearson correlation coefficients. Due to the small number of patients for each item on the m-MRC scale, the patients were categorized into 2 groups according to the m-MRC scale (<2 or ≥2); this cut-off separates ‘low breathlessness' from ‘high breathlessness' (1). The associations between the m-MRC scale (dichotomous outcome) and demographic, clinical and pulmonary function variables were determined using Student's *t*-test for independent samples, the Mann-Whitney test for non-Gaussian data and the chi-squared test for categorical data.

Forward stepwise regression analysis was used to identify the resting lung function parameters (spirometry, body plethysmography and N_2_SBW test) and possible confounders (demographic and clinical data) that were independently related to performance during the CPET (peak VO_2_ and breathing reserve) and HRQoL (CAT, SF36 PCS, SF 36 MCS, and SGRQ). The data that were tested as independent variables in the forward stepwise regression analysis were as follows: forced vital capacity (FVC), FEV_1_, FEV_1_/FVC, forced expiratory flow during the middle half of the FVC (FEF_25–75%_), TLC, RV, RV/TLC, SIII_N2_, and CV/VC. These models were adjusted for relevant confounders (sex, age, body mass index, smoking history, GOLD stages, and degree of dyspnea).

In addition, the forward stepwise regression analysis was used to identify the variables provided by spirometry and body plethysmography that were independently related to parameters of the N_2_SBW test after considering confounding factors (including demographic and clinical data). The overall performance measures of the regression models was evaluated using the coefficient of determination (R^2^), adjusted for the number of variables retained in the proposed model (cumulative R^2^).

Calibration was verified using the calibration plot (observed *vs* predicted outcomes, along with regression lines showing the slope and intercept) and the limits of agreement (LoA) plot for each of the proposed models (25).

Data were analyzed using SAS software version 6.11 (SAS Institute, Inc., USA). Differences were considered significant for P values less than 0.05.

## Results

Among the 38 patients evaluated for inclusion in this study, 7 were excluded for the following reasons: respiratory exacerbations in the month before recruitment (n=3), history of pleuropulmonary disease not associated with COPD (n=3), and use of home oxygen therapy (n=1). The average age was 67.5±9.9 years, and the average smoking load was 50.9±26.2 pack-years. All but 4 patients were treated with inhaled bronchodilators and/or inhaled corticosteroids. The demographic and clinical data, resting lung function parameters, and CPET data are shown in Table 1.

**Table 1.** Demographic and clinical data, resting lung function parameters, and cardiopulmonary exercise test data of the patients studied.

Variable	Data
Demographic characteristics	
Gender (male)	15 (48.4)
Age (years)	67.5±9.9
BMI (kg/m^2^)	22.7±4.8
GOLD stages	
1–2	18 (58.1)
3–4	13 (41.9)
A–B	17 (54.8)
C–D	14 (45.2)
Clinical data	
m-MRC scale	
<2	19 (61.3)
≥2	12 (38.7)
CAT score	18.6±11.5
SF36 PCS	46.8±19.1
SF36 MCS	57.1±10.2
SGRQ	45.1±20.6
Resting lung function	
Spirometry	
FVC (% predicted)	82.9±19.3
FEV_1_ (% predicted)	56.7±20.6
FEV_1_/FVC (%)	52.5±12
FEF_25-75%_ (% predicted)	24.3±15.5
Body plethysmography	
TLC (% predicted)	120.2±28.7
RV (% predicted)	172.4±77.4
RV/TLC (%)	55.6±13.2
Nitrogen single-breath washout test	
SIII_N2_	307.3±184.8
CV/VC	183.0±91.0
Cardiopulmonary exercise test	
Peak VO_2_ (% predicted)	52.0±19.2
RER max	1.25±0.23
O_2_ pulse max (% predicted)	64.7±20.4
HRR (beats/min)	35±17.5
Breathing reserve (%)	40.6±23.2

Data are reported as means±SD or number (%). BMI: body mass index; GOLD: Global Initiative for Obstructive Lung Disease; m-MRC: modified medical Research Council; CAT: COPD assessment test; SF36 PCS: 36-Item Short Form Health Survey-physical component summary; SF36 MCS: 36-Item Short Form Health Survey-mental component summary; SGRQ: St. George's Respiratory Questionnaire; FVC: forced vital capacity; FEV_1_: forced expiratory volume in one second; FEF_25-75%_: forced expiratory flow during the middle half of the FVC; TLC: total lung capacity; RV: residual volume; SIII_N2_: phase III slope of nitrogen single-breath washout; CV/VC: closing volume/vital capacity; peak VO_2_: peak oxygen uptake; RER max: maximum respiratory exchange ratio (VCO_2_/VO_2_) at peak exercise; O_2_ pulse max: maximum oxygen pulse (VO_2_/heart rate) at peak exercise; HRR: heart rate reserve.

**Figure 1.**
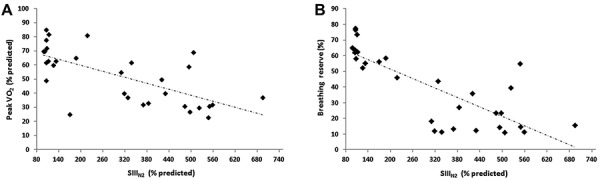
*A*, Relationship of peak oxygen uptake (peak VO_2_) with the phase III slope of nitrogen single-breath washout (SIII_N2_) (r=-0.681, P<0.0001). *B*, Relationship of breathing reserve with the SIII_N2_ (r=-0.799, P<0.0001).

The univariate correlations between the demographic, clinical, lung function and CPET data are shown in Table 2 and Figure 1. Peak VO_2_ was negatively correlated with SIII_N2_ (r=-0.681, P<0.0001) and positively correlated with FEV_1_ and FEF_25-75%_ (r=0.441, P=0.013, and r=0.393, P=0.029, respectively). Breathing reserve was negatively correlated with SIII_N2_, CV/VC, RV and RV/TLC (r=-0.799, P<0.0001; r=-0.471, P=0.007; r=-0.401, P=0.025, and r=-0.608, P=0.0003, respectively) and positively correlated with FEV_1_, FVC, FEV_1_/FVC and FEF_25-75%_ (r=0.721, P<0.0001; r=0.592, P=0.0004; r=0.670, P<0.0001, and r=0.635, P=0.0001, respectively). We further assessed the correlations between parameters of the N_2_SBW test with resting lung function variables and possible confounders. SIII_N2_ was negatively correlated with FEV_1_, FVC, FEV_1_/FVC and FEF_25-75%_ (r=-0.659, P=0.0001; r=-0.554, P=0.001; r=-0.596, P=0.0004, and r=-0.578, P=0.0007, respectively) and positively correlated with RV and RV/TLC (r=0.411, P<0.022 and r=0.548, P=0.001, respectively). CV/VC was negatively correlated with FEV_1_, FVC, FEV_1_/FVC and FEF_25-75%_ (r=-0.583, P=0.0006; r=-0.702, P<0.0001; r=-0.471, P=0.007, and r=-0.535, P=0.002, respectively) and positively correlated with age, TLC, RV and RV/TLC (r=0.464, P=0.009; r=0.648, P=0.0001; r=0.725, P<0.0001, and r=0.737, P<0.0001, respectively). Changes in the demographic, clinical, lung function and CPET variables according to the m-MRC scale (class <2 *vs* class ≥2) were also evaluated; the results of this analysis indicated no significant difference in the variables between the patients divided into the 2 classes of the m-MRC scale.

**Table 2.** Pearson's correlation coefficients for demographic, clinical, resting pulmonary function and cardiopulmonary exercise test variables.

Variables	Age	BMI	Cigarette*	FVC	FEV_1_	FEV_1_/FVC	FEF_25-75%_	TLC	RV	RV/TLC	SIII_N2_	CV/VC
**CAT**												
r	−0.102	−0.203	−0.038	**−0.412**	−0.331	−0.253	−0.189	0.323	**0.410**	**0.489**	**0.591**	0.267
P value	0.58	0.27	0.84	**0.021**	0.069	0.17	0.31	0.076	**0.022**	**0.005**	**0.0005**	0.15
**SF36 PCS**												
r	0.017	0.286	−0.138	**0.482**	**0.533**	**0.455**	**0.478**	**−0.664**	**−0.730**	**−0.664**	**−0.475**	**−0.505**
P value	0.93	0.12	0.46	**0.006**	**0.002**	**0.010**	**0.006**	**<0.0001**	**<0.0001**	**<0.0001**	**0.007**	**0.004**
**SF36 MCS**												
r	0.074	0.090	−0.065	**0.359**	0.351	0.253	0.354	0.012	−0.083	−0.171	−0.218	−0.235
P value	0.69	0.63	0.73	**0.047**	0.05	0.17	0.051	0.95	0.66	0.36	0.24	0.20
**SGRQ**												
r	−0.175	−0.191	0.126	−0.320	−0.335	−0.261	−0.308	0.193	0.311	0.188	0.122	0.156
P value	0.35	0.30	0.50	0.079	0.065	0.16	0.092	0.30	0.089	0.31	0.51	0.40
**Peak VO2**												
r	−0.021	0.076	−0.151	0.272	**0.441**	0.317	**0.393**	−0.005	−0.126	−0.175	**−0.681**	−0.025
P value	0.91	0.68	0.42	0.14	**0.013**	0.082	**0.029**	0.98	0.50	0.35	**<0.0001**	0.90
**Breathing reserve**												
r	−0.352	0.284	−0.119	**0.592**	**0.721**	**0.670**	**0.635**	−0.235	−**0.401**	−**0.608**	−**0.799**	−**0.471**
P value	0.052	0.12	0.52	**0.0004**	**<0.0001**	**<0.0001**	**0.0001**	0.20	**0.025**	**0.0003**	**<0.0001**	**0.007**

CAT: COPD assessment test; SF36 PCS: 36-Item Short Form Health Survey-physical component summary; SF36 MCS: 36-Item Short Form Health Survey-mental component summary; SGRQ: St. George's Respiratory Questionnaire; peak VO_2_: peak oxygen uptake; BMI: body mass index; FVC: forced vital capacity; FEV_1_: forced expiratory volume in 1 s; FEF_25-75%_: forced expiratory flow during the middle half of the FVC; TLC: total lung capacity; RV: residual volume; SIII_N2_: phase III slope of nitrogen single-breath washout; CV/VC: closing volume/vital capacity. *Pack-years.

We investigated whether the resting lung function variables (spirometry, body plethysmography and N_2_SBW test) could independently predict performance during the CPET and the HRQoL in these patients. The results of the forward stepwise regression analysis for the prediction of the CPET and HRQoL variables are shown in Table 3. SIII_N2_ was an independent predictor of the CAT score, peak VO_2_ and breathing reserve. RV, FVC and FEV_1_ were independent predictors of SF36 PCS, SF 36 MCS and breathing reserve, respectively. The SGRQ could not be predicted by any independent variables in the models. We further assessed whether the resting lung function variables would demonstrate an independent role in predicting the parameters of the N_2_SBW test. FEV_1_ and RV/TLC were the only independent predictors for SIII_N2_ and CV/VC, respectively (Table 3). In addition, demographic and clinical variables (including GOLD stages) were not retained in any of the proposed multiple regression models (P>0.05).

**Table 3.** Independent linear models for variables of cardiopulmonary exercise test, quality of life and single-breath washout test using resting pulmonary function variables.

Outcome variable	Independent variable^+^	B	SEB	P value	Cumulative R^2^
Cardiopulmonary exercise test*					
Peak VO_2_	SIII_N2_	−0.071	0.014	<0.0001	0.46
Breathing reserve	SIII_N2_	−0.072	0.017	0.0002	0.71
	FEV_1_	0.387	0.153	0.017	
Quality of life*					
CAT	SIII_N2_	0.037	0.009	0.0004	0.35
SF36 PCS	RV	−0.180	0.031	<0.0001	0.53
SF36 MCS	FVC	0.191	0.092	0.047	0.13
Single-breath washout test#					
SIII_N2_	FEV_1_	−5.911	1.253	0.0001	0.43
CV/VC	RV/TLC	5.100	0.868	<0.0001	0.54

B: regression coefficient; SEB: standard error of the regression coefficient; peak VO_2_: peak oxygen uptake; SIII_N2_: phase III slope of nitrogen single-breath washout; FEV_1_: forced expiratory volume in one second; CAT: COPD assessment test; SF36 PCS: 36-Item Short Form Health Survey-physical component summary; RV: residual volume; SF36 MCS: 36-Item Short Form Health Survey-mental component summary; FVC: forced vital capacity; CV/VC: closing volume/vital capacity; TLC: total lung capacity. *Forward stepwise regression models using resting pulmonary function variables (spirometry, body plethysmography and N_2_SBW test. ^#^Forward stepwise regression analysis using resting pulmonary function variables (spirometry and body plethysmography). Models were adjusted for confounders (demographic and clinical data). ^+^Demographic and clinical variables were not retained in any of the forward stepwise regression models shown above (P<0.05).

### Calibration

**Figure 2.**
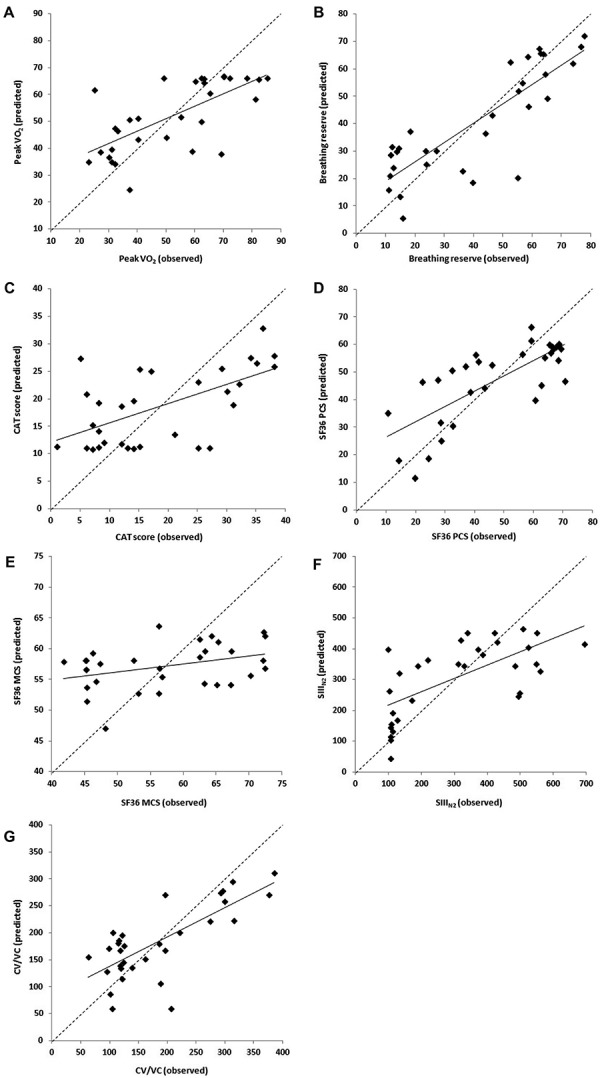
Calibration plots of the observed versus predicted values. *A*, Analysis of the multiple linear regression models for peak oxygen uptake (peak VO_2_); *B*, breathing reserve; *C*, COPD assessment test (CAT); *D*, 36-Item Short Form Health physical component summary (SF36 PCS); *E*, 36-Item Short Form Health Survey-mental component summary (SF36 MCS); *F*, phase III slope of nitrogen single-breath washout (SIII_N2_); and *G*, closing volume/vital capacity (CV/VC).

**Figure 3.**
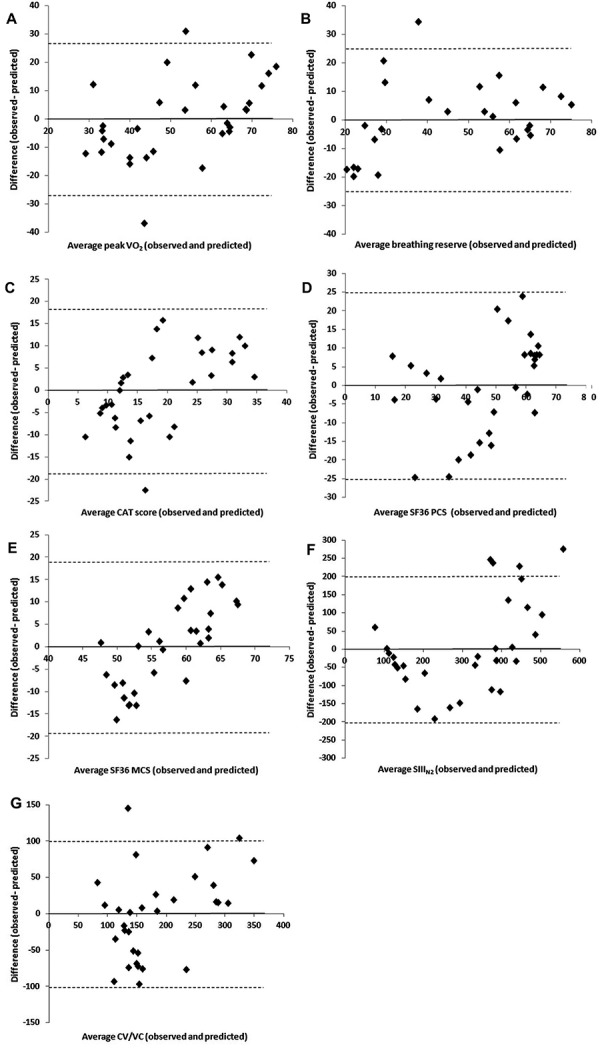
Limits of agreement plots of the averaged values and the differences (observed - predicted values). *A*, Analysis of the multiple linear regression models for peak oxygen uptake (peak VO_2_); *B*, breathing reserve; *C*, chronic obstructive pulmonary disease assessment test (CAT); *D,* 36-Item Short Form Health Survey-physical component summary (SF36 PCS); *E*, 36-Item Short Form Health Survey-mental component summary (SF36 MCS); *F*, phase III slope of nitrogen single-breath washout (SIII_N2_); and *G*, closing volume/vital capacity (CV/VC).

Regarding the models for peak VO_2_, breathing reserve, CAT, SIII_N2_ and CV/VC, the following findings were observed: there were no obvious relationship between the differences and the means given by the lines (Figure 2); most of the differences occurred within the LoA with a random distribution over the mean (Figure 3), and the histograms of the residues showed an approximately normal distribution and absence of evident asymmetry. On the contrary, the models for SF36 PCS and SF36 MCS did not present satisfactory calibrations.

### Power analysis

To provide a context for interpreting the null findings, a *post hoc* power calculation was done based on the actual sample size (n=31) and the observed correlation for the main outcome (peak VO_2_) and SIII_N2_ (r=-0.681). For an alpha of 0.05, the power was 99.3%, showing the adequacy of the studied sample size to get significant results.

## Discussion

The main finding of this study was that, in patients with COPD, a greater inhomogeneity in ventilation corresponded to a lower oxygen consumption and breathing reserve at peak exercise. The inhomogeneity of ventilation was also positively associated with poor HRQoL of these patients. Compared with the heterogeneity in ventilation, the changes in small airways measured by the N_2_SBW test were associated to a lesser extent with the CPET parameters and HRQoL. In addition, other lung function variables measured by spirometry and body plethysmography were associated with performance during exercise and HRQoL of patients with COPD. To our knowledge, this study is the first to demonstrate that the parameters of the N_2_SBW test partially explain the exercise intolerance and poor quality of life in stable COPD patients.

Although physical limitations to exercise are undoubtedly multifactorial, ventilatory factors are often the main limitation for poor performance during exercise in patients with COPD (13). The increased ventilatory demand in these patients is primarily caused by inefficient ventilation. In the present study, we observed that SIII_N2_ (but not the CV/VC ratio) was the only independent predictor of peak VO_2_. Interestingly, Jones et al. (26) evaluated 19 patients with COPD with mild to moderate airflow obstruction and found that those with low peak VO_2_ had a higher ventilatory inefficiency (higher ventilation/carbon dioxide output nadir) during the CPET. Therefore, we believe that increased SIII_N2_ at rest indirectly indicates the ventilatory inefficiency detected during symptom-limited maximal exercise. Of note, our models were adjusted for relevant confounders (including the anthropometric data, demographic variables and GOLD stages). Furthermore, we used the predicted values for the lung function parameters, which adjust the absolute values for gender, age, weight and height (18,25).

In our study, the strongest correlation was observed between SIII_N2_ and breathing reserve measured at peak exercise (r=-0.799, P<0.0001). Breathing reserve depends on several factors that are responsible for ventilatory demand, including metabolic demand, body mass, dead space ventilation and neuroregulatory mechanisms (10). A low breathing reserve suggests that the individual's exercise capacity may be limited by ventilatory capacity. Jones et al. (26) evaluated patients with mild to moderate COPD and observed that the ventilation "wasted" in emphysematous areas (but not small airway disease) was associated with a decrease in ventilatory efficiency in the CPET. This result explains, at least in part, the finding that the increase in SIII_N2_ is strongly correlated with the depletion of ventilatory reserve observed in our study and that SIII_N2_ is independent of the peak VO_2_ and the degree of airflow obstruction. In fact, there is mounting evidence that COPD patients with relatively well-preserved FEV_1_ may exhibit substantial emphysema in computed tomography (CT) and that these abnormalities are associated with significant patient-centered outcomes, including shortness of breath and exercise intolerance (27,28). Emphysema increases air spaces, decreases alveolar attachments to the small airways and impairs microvascular perfusion, which together cause a significant maldistribution of ventilation (26,29).

In contrast to the variations in SIII_N2_, our results indicate that changes in the CV/VC may not be a major contributor to CPET measurements in patients with COPD because CV/VC was not retained in any of the regression models that were developed. In the N_2_SBW test, an increased CV/VC reflects air trapping because of small airway closure, and therefore, it can potentially provide information about early abnormalities in the small airways in COPD (30). Although small airways may negatively affect the total airway resistance and restrict the distribution of ventilation to some extent, the changes in lung mechanics from small airway abnormalities in many of these patients are mild (31).

Although COPD is primarily a lung disease, it also causes significant systemic effects that can lead to a decline in health status and HRQoL (32). A recent meta-analysis indicated that CAT could be used as a complementary tool in the clinical assessment of patients to predict exacerbations of COPD, deterioration in health status, depression, and mortality (33). Only 3 studies involving 444 patients had described regression models that predicted the CAT score using clinical, spirometry and body plethysmography variables and did so with R^2^ values that explained less than 50% of the variance ([Bibr B34] [Bibr B35] 3436). Considering that the heterogeneity of ventilation was not evaluated in any of these studies, we believe that this parameter is important because in our study SIII_N2_ alone explained 35% of the variability of the CAT scores. The clinical variables and traditional PFTs were not retained in the regression models in our study, likely because of the strong association between CAT and SIII_N2_. In contrast to CAT, we were unable to build a regression model for the m-MRC scale. This result was expected because the CAT is a multidimensional scale that includes seven other items, not only shortness of breath, whereas the m-MRC scale is one-dimensional and assesses only dyspnea (33).

In this study, the SF36 PCS was associated with various physiological parameters at rest. However, the strongest correlation was observed between the increase in RV and the decrease in SF36 PCS, and RV was the only physiological parameter that was included in the SF36 PCS regression model. Air trapping caused by the combined effect of peripheral airway disease and increased ventilatory demand imposes critical mechanical restrictions and worsens dyspnea, which, in turn, leads to the decline of HRQoL of patients with COPD (13). Interestingly, after evaluating patients with heterogeneous emphysema who underwent lung volume reduction surgery, Oey et. (37) observed a significant reduction in air trapping that was proportional to the improvement in the HRQoL measured by the SF-36 PCS.

Our findings were similar to those of Morishita-Katsu et al. (36) who evaluated 109 subjects with stable COPD and observed a weak correlation between the SGRQ score and clinical and functional variables at rest. Regarding exercise, Mirdamadi et al. (38) evaluated 37 patients with COPD (GOLD I-III) and found no significant correlation between peak VO_2_ and the SGRQ score. Similarly, other researchers reported that peak VO_2_ is a poor indicator of health status (3,39), suggesting that CPET parameters at peak exercise may not be effective for evaluating the ADLs of patients. Although specific for COPD, the SGRQ is an extensive test that is often stressful to the patient, and it measures health status using several parameters, which might, at least in part, be responsible for the lack of correlation with clinical and physiological data (30).

Our study is characterized by the following limitations. First, this study included a small number of subjects from a single center and employed a cross-sectional design; therefore, the temporal effect on the stability of the response of each evaluated tool is unknown. Due to the multiple statistical tests performed, some of the findings may be a result of chance. Therefore, there is the possibility of type I error, i.e., accepting an alternative hypothesis when the results can be attributed to chance (40). Second, chest CT could help elucidate the maldistribution of ventilation, as there is growing evidence that emphysema is associated with low exercise tolerance in COPD, even in patients with mild to moderate airflow obstruction (26). Third, we did not assess important findings of COPD patients in the regression models studied, including socioeconomic status, comorbidities, and pharmacologic therapy, which could have allowed us to obtain more robust models. Finally, the lung function variables are reported in relation to the predicted values, and thus, these variables were corrected for the anthropometric and demographic data; this at least partially explains why the anthropometric and demographic data were not retained in the proposed models. Despite these limitations, our study provides valuable information for N_2_SBW test assessments in randomized controlled trials. In this context, the N_2_SBW test may contribute to the categorization of patients and the evaluation of responses to various clinical and surgical therapies for COPD.

In conclusion, there was a relationship between N_2_SBW test measurements and CPET parameters in stable COPD patients. In these patients, inhomogeneity of ventilation explains much of the exercise intolerance assessed by the CPET and, to a lesser extent, the poor HRQoL. Compared with the N_2_SBW test, the variables measured by spirometry and body plethysmography more strongly influenced the HRQoL and, to a lesser degree, oxygen consumption in patients with COPD. The contribution of the N_2_SBW test to COPD studies should be further explored, as this test has the potential to be used as a complementary tool in clinical practice to assess these patients.
